# Review of Oxides Prepared by a Short Process Using Rare-Earth Chlorides

**DOI:** 10.3390/ma18204669

**Published:** 2025-10-11

**Authors:** Jing Wei, Xue Bian, Xinmiao Zhu, Hao Huang, Chunlin Ye, Shuchen Sun, Liqin Zhong, Ganfeng Tu

**Affiliations:** 1 Key Laboratory for Ecological Metallurgy of Multimetallic Mineral (Ministry of Education), Northeastern University, Shenyang 110819, China; 2 School of Metallurgy, Northeastern University, Shenyang 110819, China; 3 China Rare Earth Group Jiangxi Co., Ltd., Ganzhou 341000, China

**Keywords:** fixed-valence rare-earth, variable-valence rare-earth, rare-earth oxide, pyrolysis, industrial application

## Abstract

Direct thermal decomposition of rare-earth chlorides into rare-earth oxides (REOs) in a single step presents a short-process, wastewater-free, and environmentally friendly alternative to the conventional precipitation–calcination method, which produces large amounts of saline wastewater. While earlier reviews have primarily focused on summarizing reaction conditions and thermodynamic parameters, they have seldom discussed the critical variations in pyrolysis behavior across different rare-earth elements. This review highlights a novel classification of rare-earth chlorides into fixed-valence and variable-valence groups, revealing how their respective oxidation states govern thermodynamic stability, reaction pathways, and chlorine release behavior. Furthermore, a systematic comparison is provided on the effects of additives, temperature, and gas partial pressure on product purity, particle size, and microstructure, with particular attention to the mechanisms underlying oxychloride intermediate formation. Beyond fundamental reaction principles, this work uniquely evaluates the design and performance of existing pyrolysis reactors, outlining both opportunities and challenges in scaling up direct rare-earth chloride (RECl_x_) pyrolysis for industrial REO production. By integrating mechanistic insights with reactor engineering considerations, this review offers advancements over previous descriptive summaries and proposes a strategic pathway toward sustainable rare-earth processing.

## 1. Introduction

**Figure 1:**
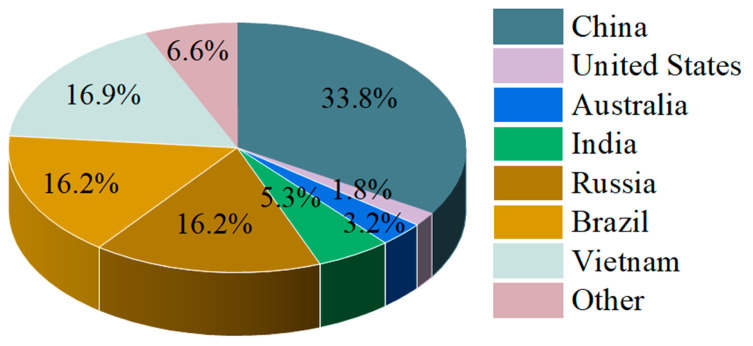
The distribution of global rare-earth resources according to deposit types and countries of origin [6].

Rare-earth elements (REEs) are critical strategic metal resources [1,2] and play indispensable roles in high-tech civilian industries and defense technologies [3,4]. According to the United States Geological Survey (USGS), global rare-earth reserves are estimated at approximately 130 million tons, with the majority concentrated in a few countries, namely China, Brazil, Vietnam, and Russia [5]. China possesses the largest share, with 44 million tons (33.8% of the global total), followed by Vietnam with 22 million tons, and Brazil and Russia each with 21 million tons [5]. Collectively, these four countries account for over 83% of worldwide rare-earth reserves, reflecting a highly concentrated resource distribution (Figure 1).

The industrial production chain for REEs generally consists of the following sequential steps: mining of ores or recycling of secondary resources, decomposition of concentrates or primary oxides, solvent extraction and separation, precipitation of rare-earth salts, calcination to produce rare-earth oxides (REOs), electrolytic reduction to metals, and finally fabrication into advanced materials [7,8,9]. Among them, REOs serve not only as precursors for metallic rare earths but also find important applications in polishing materials, ceramics, and luminescent devices [10,11,12,13,14]. The traditional method for REO production involves chemical precipitation followed by high-temperature calcination [15,16]. This process consumes 1.2 to 1.5 times the theoretical amount of precipitating agents such as ammonium bicarbonate or oxalic acid and generates wastewater with chloride concentrations ranging from 3 to 40 g/L [17,18]. Wastewater discharge can reach 1.5 to 6 million cubic meters per 10,000 tons of REOs produced [19]. Therefore, there is a pressing need to develop environmentally friendly and resource-efficient processes for converting rare-earth chlorides into oxides.

The pyrolysis mechanism entails coupled thermodynamics and kinetic processes, including, solvent evaporation, solute precipitation, solute decomposition, and oxide crystallization within the solution aerosol.

Spray pyrolysis (SP), which emerged in the 1980s [20], evolved from spray drying (SD). A key distinction is that SP involves simultaneous solvent evaporation and solute decomposition [21]. Building on the Ruthner process originally devised for acid recovery in the steel industry [22,23,24], SP has become a versatile technique for synthesizing ultrafine REOs. To improve the quality of iron oxide products, researchers have optimized furnace designs and process parameters, significantly broadening the applications of spray roasting [24,25,26,27,28,29]. Furthermore, numerical simulations of ferrous chloride decomposition in industrial-scale spray roasting reactors have laid a theoretical foundation for process optimization [30,31,32,33].

In the early 20th century, thermal decomposition of metal chloride solutions was employed to produce magnesia from brucite. However, limited understanding of gas-liquid-solid interactions, initially led to crust formation of aqueous magnesium chloride on furnace walls. This issue was later resolved through optimized process parameters furnace design, and adoption of the Ruthner spray-roasting technique [34]. In pyrolysis of MgCl_2_-laden industrial wastewater, adding H_2_O_2_ promotes chlorine removal via the oxidative action of H_2_O_2_ or in situ-generated O_2_, thereby lowering both the decomposition temperature and energy consumption [35,36]. Beyond iron and magnesium oxides, spray calcination has also been used to produce Co_3_O_4_ and NiO powders [37,38]. In 2014, researchers directly utilized aluminum chloride solutions in SP to synthesize alumina [39]. The chlorine content of alumina produced under a steam atmosphere was significantly lower than that under argon [40]. In 2019, Shibayama et al. [41] systematically investigated the pyrolysis of NiCl_2_, CoCl_2_, and FeCl_2_, elucidating their decomposition mechanisms.

For REOs, SP studies have mainly employed chloride and nitrate solutions as precursors [42,43,44,45,46,47,48,49,50,51]. However, decomposition pathways and particle-formation mechanisms differ considerably between these salts. Nitrate pyrolysis proceeds rapidly with minimal elemental segregation, rendering it well-suited for multicomponent ultrafine powder synthesis [48,49,50,51]. However, rare-earth nitrates are not direct products of rare-earth smelting; they must be prepared by dissolving rare-earth carbonates or oxides in nitric acid, thereby increasing overall production costs. In contrast, rare-earth chloride solutions are direct intermediates in the extraction and separation process. Adopting SP for direct REO, production could reduce precipitant usage by 0.7–0.8 t/t of REO and lower CO_2_ emissions by 0.3–0.6 t/t REO. Owing to the diversity in REE types and valence states, the mechanisms and conditions for thermal decomposition of different rare-earth chlorides vary significantly. Studies have demonstrated that cerium chloride, which has a relatively low theoretical decomposition temperature, can be directly pyrolyzed to form cerium oxide. Conversely, lanthanum chloride, with a higher decomposition temperature, predominantly forms lanthanum oxychloride when pyrolyzed at around 1200 °C. However, introducing hydrogen peroxide (H_2_O_2_) markedly reduces the pyrolysis temperature of lanthanum chloride and promotes the formation of lanthanum hydroxide [52]. Moreover, using praseodymium-neodymium chlorides, which possess intermediate decomposition temperatures, as precursors in ultrasonic SP synthesis of gadolinium oxide and praseodymium-neodymium oxides, along with hydrated citric acid as a pyrolysis aid, substantially enhances the performance of the resulting oxides [53].

To date, comprehensive summaries and analyses of the technology and equipment for the direct pyrolysis of all seventeen rare-earth chlorides into oxides have been lacking. This gap has hindered large-scale application despite growing industrial interest. Accordingly, this paper categorizes REEs into fixed-valence and variable-valence groups. It reviews recent advances in pyrolysis processes, reaction mechanisms, product characteristics, and reactor designs, and analyzes challenges in process optimization and application. The aim is to provide a reference for future research and industrialization efforts.

## 2. Research the Status of the Pyrolysis Process for Rare-Earth Chlorides

REOs exhibit considerable application potential in thin films [54], luminescent materials [55], and battery materials [56,57]. The preparation parameters and applications of REOs vary significantly depending on the valence states of the constituent REEs. In this review, REEs are categorized based on their predominant oxidation states: most lanthanides primarily exist in the trivalent state and are classified as “constant-valence” elements, whereas cerium, praseodymium, and terbium can stabilize higher oxidation states (e.g., +4), marking them as “variable-valence” elements. Although neodymium may form divalent compounds under specific conditions, its chemistry is dominated by the trivalent state, supporting its inclusion among the constant-valence elements. Accordingly, this review separately summarizes and analyzes the spray-calcination pyrolysis behavior of these two categories of rare-earth chlorides.

### 2.1. Pyrolysis of Fixed-Valence Rare-Earth Chlorides

Rare-earth chlorides are prone to hydrolysis, leading to the formation of oxychlorides, and their complete conversion to oxides generally requires high temperatures. For instance, static pyrolysis of LaCl_3_ and NdCl_3_ solutions at 1100 °C yields mainly LaOCl and NdOCl [58,59]. Similarly, the thermal decomposition of GdCl_3_·6H_2_O involves hydrolysis, resulting in GdOCl [60]. Since oxychloride formation is often unavoidable, direct pyrolysis presents a challenge for producing high-purity REOs.

1. Lanthanum (La)

**Figure 2:**
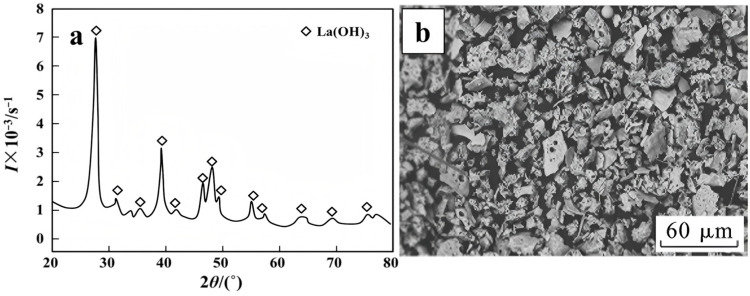
(**a**) XRD analysis of the product, (**b**) SEM analysis of La(OH)_3_ product [52].

During the thermal decomposition of LaCl_3_·7H_2_O, dehydration occurs initially [61,62], followed by pyrolysis at elevated temperatures. The theoretical temperature required for the direct conversion of hydrated lanthanum chloride to lanthanum oxide is 1180 °C. Under typical pyrolysis furnace conditions, the primary product is therefore lanthanum oxychloride (LaOCl). To overcome this high-temperature requirement, the addition of hydrogen peroxide to the lanthanum chloride solution before pyrolysis promotes the formation of lanthanum hydroxide. The corresponding reaction (Equation (1)) proceeds at a theoretical temperature of only 288 °C. Pyrolysis experiments coupled with XRD analysis confirmed a La(OH)_3_ conversion rate of 99.96% (Figure 2a), while SEM imaging revealed a fragmented particle morphology (Figure 2b) [52]. This approach is applicable to various rare-earth chlorides [63]. The resulting lanthanum hydroxide can be subsequently calcined to form nano-sized lanthanum oxide, optionally via the intermediate LaOOH [64], offering an energy-efficient and environmentally friendly pathway for low-temperature REO preparation.

2LaCl_3_(l) + 3H_2_O_2_(l) = 2La(OH)_3_(s) + 6HCl(g) (1)

Alternatively, a sub- or super-critical steam pyrolysis route has been proposed for REO synthesis. In this process, lanthanum chloride crystals are first dried in a hot-air oven, then treated with high-pressure steam in a reactor at elevated temperature and pressure. The product obtained under these conditions is La_2_O_3_ with a particle size of 0.22 μm and a chlorine content of 37 ppm. This method offers short process flow, high product quality, and environmental benefits. However, the demand for high-temperature, high-pressure equipment and the associated capital investment limit its industrial scalability [65]. Jin [58] investigated jet pyrolysis (JP), noting that under high-temperature conditions, the LaCl_3_ solution and hot gas undergo rapid vaporization, forming dispersed bubbles. This creates a substantially larger reaction interface compared to conventional static pyrolysis, enabling rapid heating of reactants and effectively suppressing LaOCl by-product formation.

2. Gadolinium (Gd)

**Figure 3:**
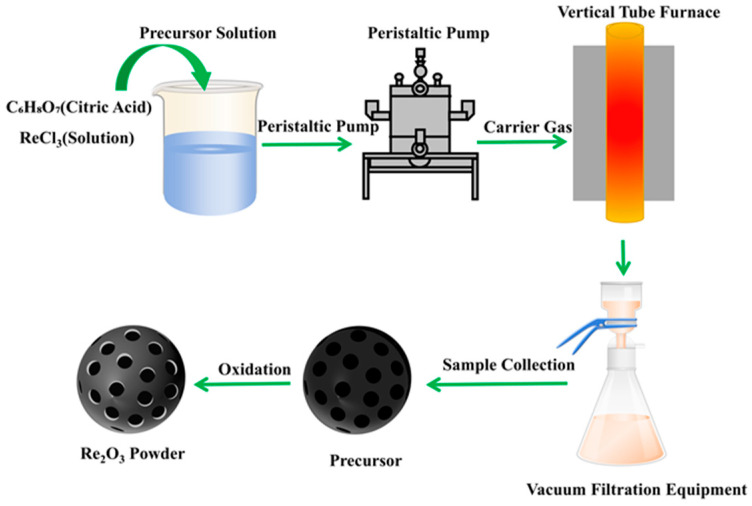
Schematic diagram of the ultrasonic SP process for the production of REO [66].

**Figure 4:**
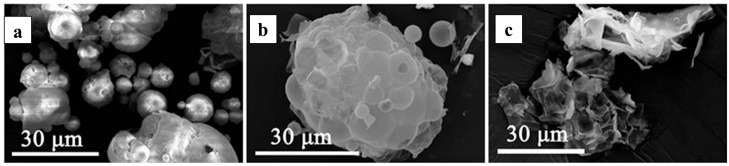
SEM images of the products [53]: (**a**) without citric acid addition; (**b**) citric acid: GdCl_3_·H_2_O = 2:1; (**c**) citric acid: GdCl_3_·H_2_O = 1:1.

Thermodynamic calculations indicate that the direct pyrolysis of gadolinium chloride to gadolinium oxide requires a theoretical temperature exceeding 1100 °C [53]. Experimentally, it has been demonstrated that adding citric acid to gadolinium chloride leads to the initial formation of carbon-containing precursors during pyrolysis. Gadolinium oxide is subsequently obtained after secondary calcination at 700 °C, significantly reducing the overall conversion temperature [53]. Fu et al. [66] successfully prepared gadolinium oxide using an ultrasonic SP process, as illustrated in Figure 3. During the pyrolysis of gadolinium chloride, the phase transformation follows the sequence GdCl_3_→Gd(OH)_2_Cl→GdOCl→Gd_2_O_3_, with the corresponding reactions given in Equations (2)–(7). The heat released from the oxidation of carbon derived from decomposed citric acid promotes the conversion of GdOCl to Gd_2_O_3_. Moreover, the presence of citric acid contributes to a smaller particle size, increased powder porosity, and a reduction in the overall pyrolysis temperature. After secondary roasting at 950 °C with citric acid, the product morphology transitions from an agglomerated to a fragmented state (Figure 4), which further enhances porosity and facilitates chlorine removal. Ultimately, the chlorine content is reduced to below 500 ppm.

2GdCl_3_(s) + 3H_2_O(g) = Gd_2_O_3_(s) + 6HCl(g) (2)

GdCl_3_(s) + H_2_O(g) = GdOCl(s) + HCl(g) (3)

GdCl_3_(s) + 2H_2_O(g) = Gd(OH)_2_Cl(s) + 2HCl(g) (4)

Gd(OH)_2_Cl(s) = GdOCl(s) + H_2_O(g) (5)

GdOCl(s) + O_2_(g) = 2Gd_2_O_3_(s) + 2Cl_2_(g) (6)

2GdOCl(s) + H_2_O(g) = Gd_2_O_3_(s) + 2HCl(g) (7)

3. Neodymium (Nd)

Research on the pyrolysis of NdCl_3_ remains limited. The theoretical temperature required for the direct conversion of NdCl_3_ to Nd_2_O_3_ is 1000 °C [66]. However, under static calcination conditions at 1100 °C, NdOCl persists as the dominant phase [67]. When citric acid is introduced during SP, C-type (cubic) Nd_2_O_3_ forms at 950 °C. With further increases in temperature, the diffraction peaks of A-type (hexagonal) Nd_2_O_3_ intensify. Owing to the carbon-rich and loosely porous morphology of the citric-acid-assisted precursor, the decomposition temperature of NdOCl is significantly reduced. At 1050 °C, the residual chlorine content of the resulting product reaches 153 ppm, well below the industrial standard of 500 ppm [66]. In an alternative approach to lower the reaction temperature, JP was employed. At 1100 °C, the main product was Nd_2_O_3_, though minor amounts of NdOCl were still detected. JP further reduced the pyrolysis temperature of NdCl_3_ to 800 °C, enabling the low-temperature formation of Nd_2_O_3_ [58].

4. Rare-Earth Composite Oxides

Wang et al. [68] synthesized γ-Al_2_O_3_/LaAlO_3_ by calcining a mixed LaCl_3_–AlCl_3_ solution with citric acid as an additive at 800 °C. During pyrolysis, the precursor LaOCl·Al_2_O_3_ first formed according to Equation (8). Subsequent calcination at 800 °C produced LaAlO_3_ with a purity exceeding 99.9% via Equation (9) [69]. The same method was also applied to synthesize LaCrO_3_ and LaFeO_3_, while γ-Al_2_O_3_ was further obtained at 1000 °C [70]. In a related study, γ-Al_2_O_3_/CeO_2_ was prepared by SP using AlCl_3_ and CeCl_3_ as starting materials [71]. In addition, spherical mixed REO particles (e.g., Nd_2_O_3_) have been directly synthesized from rare-earth leachates via ultrasonic SP [72].

2LaCl_3_(l) + 2AlCl_3_(s) + 5H_2_O = 2LaOCl(s) + Al_2_O_3_(s) + 10HCl(g) (8)

4LaOCl(s) + 2Al_2_O_3_(s) + O_2_(g) = 4LaAlO_3_ + 2Cl_2_(g) (9)

### 2.2. Pyrolysis of Variable-Valence Rare-Earth Chlorides

Variable-valence REOs are widely employed in luminescent, soft-magnetic, catalytic, and other functional materials because of their abundant oxygen vacancies. Research on preparing oxides from variable-valence rare-earth chlorides via SP technology first emerged in the early 1960s. In 1987, General Electric (GE) in the United States produced nanoscale CeO_2_ for the first time by spray-drying an aqueous cerium nitrate solution [73]. A decade later, Vallet-Regí et al. [74] also synthesized CeO_2_ nanoparticles using SP.

1. Cerium (Ce)

**Figure 5:**
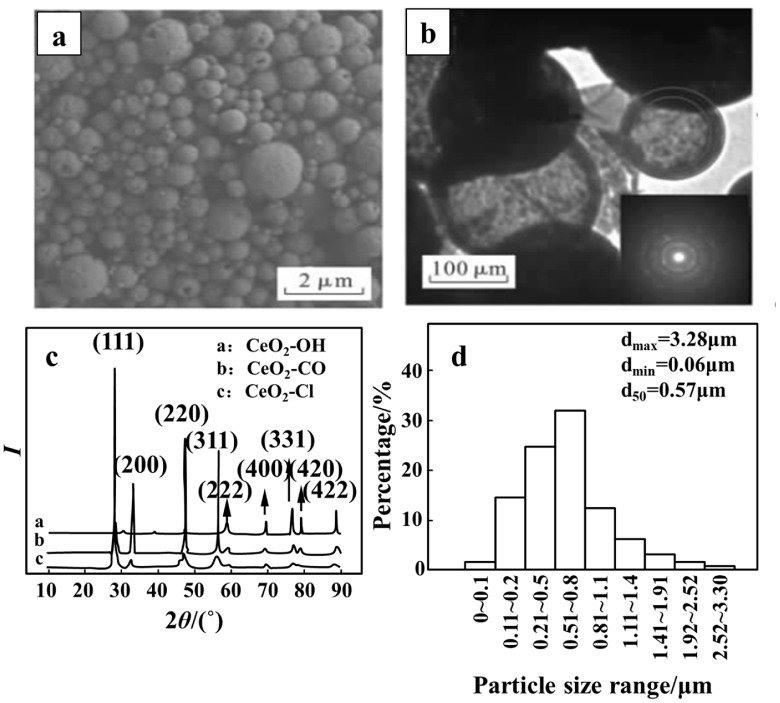
Microstructure characterization of CeO_2_ [85]: (**a**) SEM image of CeO_2_-Cl; (**b**) TEM image of CeO_2_-Cl; (**c**) XRD pattern; (**d**) Particle size statistical histogram of CeO_2_-Cl.

**Figure 6:**
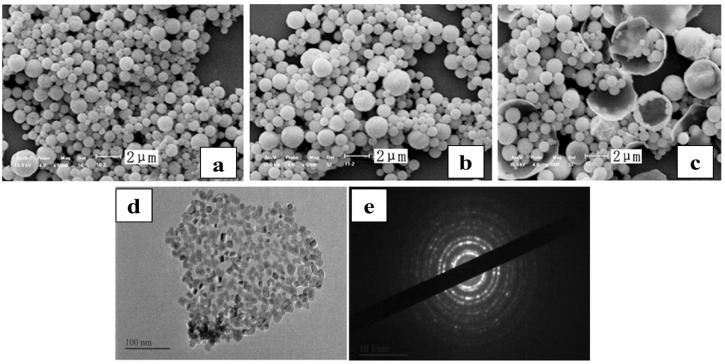
Microstructural characterization of CeO_2_: (**a**) 0.02 mol/L citric acid; (**b**) 0.029 mol/L citric acid; (**c**) 0.04 mol/L citric acid [83]; (**d**) Typical TEM image (A/C = 1.5); (**e**) SAED diffraction rings (A/C = 1.5) [82].

Studies on cerium chloride solutions have shown that dehydration occurs between 25 and 224 °C under an air atmosphere, hydrolysis proceeds from 170 to 460 °C, and oxidation reactions, as represented by Equation (10), initiate at 460 °C. However, the actual temperature required for static pyrolysis to yield CeO_2_ is as high as 700 °C [75]. Wu et al. [76] developed an ultrasonic SP route for converting industrial-grade CeCl_3_·7H_2_O into CeO_2_. As shown in Figure 5, the resulting CeO_2_ consists of hollow spherical particles with a large surface area and a size distribution of 0.06–3.28 μm, composed of polycrystalline nanocrystals. This one-step process offers a short flowsheet, high product purity, and efficient recovery of by-product HCl/Cl_2_. However, the as-prepared powders often contain broken shells or isolated hollow spheres, which impair particle uniformity and complicate subsequent applications. Further studies have shown that adding citric acid improves the sphericity and polycrystallinity of CeO_2_ (Figure 6c), yielding specific surface areas up to 59.72 m^2^/g and primary crystallite sizes as small as 15 nm [21,77,78,79,80,81,82], with negligible impact on CeO_2_ yield [83]. Other researchers have used cerium nitrate solutions in SP to obtain CeO_2_ nanoparticles with tailored morphologies and structures [84]. JP has also been shown to produce pure CeO_2_ at 500 °C, indicating that lower processing temperatures are feasible [58]. Together, these findings highlight the potential of low-temperature SP of rare-earth chloride solutions for producing morphology- and structure-controlled oxides, with promising prospects for practical application.

2CeCl_3_ + 2H_2_O + O_2_ → 2CeO_2_ + 4HCl + Cl_2_ (10)

2. Terbium (Tb)

**Figure 7:**
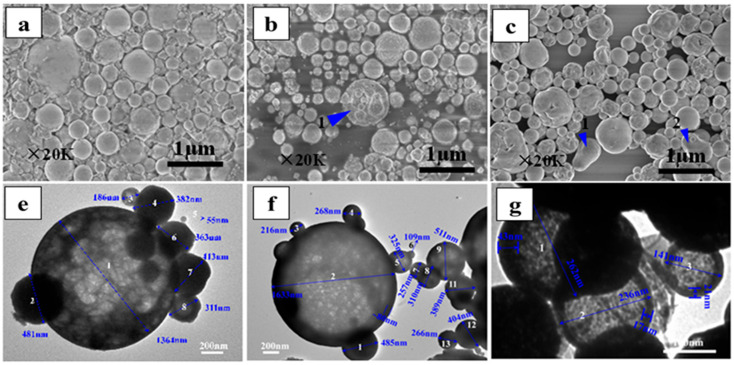
FESEM and HRTEM images of REO: (**a**,**e**) terbium oxide, (**b**,**f**) praseodymium oxide, (**c**,**g**) cerium oxide [85,86].

Xue et al. [85,86] synthesized micro-/nano-sized TbO_2_ powders via ultrasonic SP using TbCl_3_ aerosol as the precursor. The fine aerosol droplets generated by ultrasonic atomization of the rare-earth chloride solution were found to enhance pyrolysis kinetics, thereby improving the oxide conversion rate [87]. The resulting spherical TbO_2_/Tb_7_O_12_ particles exhibited diameters ranging from 0.1 to 1.3 μm, with a d_50_ of approximately 0.5 μm. The principal reactions involved in the pyrolysis of terbium chloride are summarized in Equations (11)–(13). Corresponding Field Emission Scanning Electron Microscopy (FESEM) and High-Resolution Transmission Electron Microscopy (HRTEM) images of the terbium oxide products are presented in Figure 7.

TbCl_3_ + 2H_2_O = Tb(OH)_2_Cl + 2HCl (11)

2TbOCl + O_2_ = 2TbO_2_ + Cl_2_ (12)

14TbOCl + 5O_2_ = 2Tb_7_O_12_ + 7Cl_2_ (13)

3. Praseodymium (Pr)

**Figure 8:**
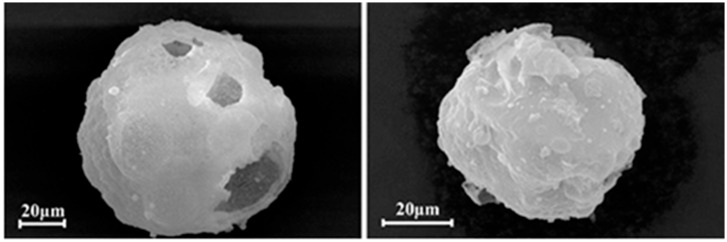
Morphology of praseodymium oxide products obtained with the addition of citric acid monohydrate [53].

During the pyrolysis of PrCl_3_, PrOCl forms initially. Thermodynamic analysis indicates that converting PrOCl to PrO_2_ requires a theoretical temperature of approximately 1400 °C. To reduce this temperature, Fu et al. [53,66] introduced citric acid as an additive and calcined the precursor statically at about 1050 °C for 4 h, yielding Pr_4_O_7_ with a residual chloride content of 136 ppm. When the calcination temperature was raised to 1200 °C, phase-pure A-type Pr_2_O_3_ was obtained. The incorporation of citric acid monohydrate promoted the formation of porous spherical shells or fragmented structures with loose morphologies (Figure 8), which improved the diffusion of reactant and product gases.

### 2.3. Rare-Earth Chloride Pyrolysis Process

Spray calcination pyrolysis allows control over oxide particle size, morphology, and purity by adjusting key parameters such as temperature, duration, and precursor concentration. Although the pyrolysis of light rare-earth chlorides (e.g., La, Ce, Pr) has been widely investigated, with corresponding parameters and product characteristics summarized in Table 1, the behavior of medium and heavy rare-earth chlorides (e.g., Ho, Dy, Er) remains largely unexplored. Future research should focus on (1) constructing temperature–time–composition phase diagrams for rare-earth chloride systems and (2) applying in situ diagnostic techniques (e.g., Thermogravimetric Analysis-Mass Spectrometry (TGA-MS), XRD, high-speed imaging) to elucidate decomposition pathways, thereby addressing the current knowledge gap in medium and heavy rare-earth chloride pyrolysis.

## 3. Pyrolysis Mechanism of Rare-Earth Chlorides

### 3.1. Thermodynamic Mechanism of the Pyrolysis Process

**Figure 9:**
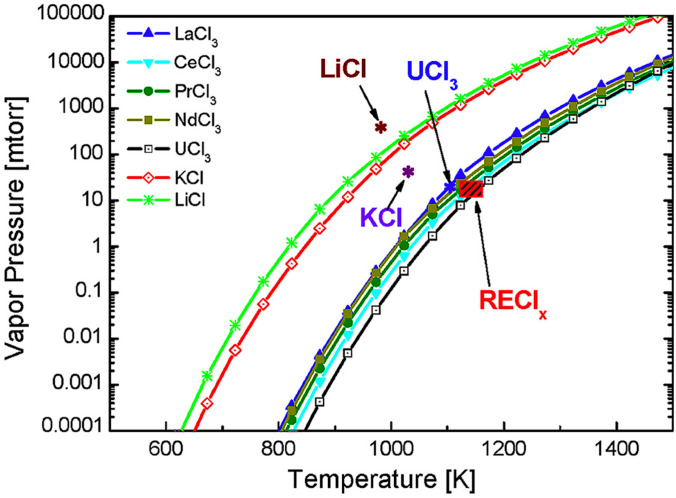
Thermodynamic dependence of the vapor pressures of different chlorides [89].

The thermal decomposition of metal chloride solutions into oxides proceeds primarily through hydrolysis reactions, with the partial pressures of H_2_O and HCl, as well as the reaction temperature, serving as the main controlling factors [40,88]. Thermodynamic calculations of the temperature-dependent vapor pressure of various chlorides [89] (Figure 9) reveal that the pyrolysis sequence differs at a given temperature, following the order: LiCl < KCl < UCl_3_ < RECl_x_ [89]. Rare-earth chlorides, in particular, exhibit strong temperature dependence and generally require high temperatures and extended residence times for complete decomposition. However, the rapid reaction kinetics in SP partly compensate for this limitation.

1. Pyrolysis Temperature

**Figure 10:**
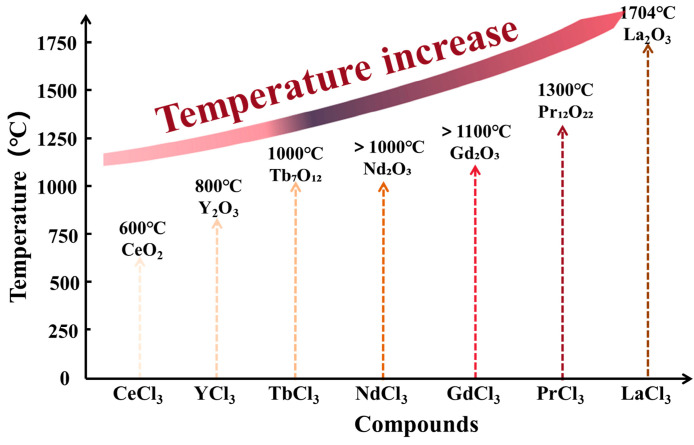
Decomposition sequence of rare earth chlorides.

Temperature is a critical parameter in the pyrolysis of rare-earth chlorides to their corresponding oxides, significantly influencing both the reaction pathway and the final oxide phase composition. For instance, LaCl_3_ requires an extremely high temperature of approximately 1704 °C for complete conversion to La_2_O_3_ [52]. In contrast, CeCl_3_ is fully pyrolyzed to CeO_2_ between 600 and 1000 °C in air [76], indicating its relatively low thermal stability among rare-earth chlorides. PrCl_3_ pyrolyzed at 1300 °C yields Pr_12_O_22_ as the main product [85,87]. The conversion of NdCl_3_ to Nd_2_O_3_ requires a minimum temperature of 1000 °C; under SP conditions, however, NdOCl tends to form as an intermediate and remains stable until temperatures exceed 2200 °C [66]. GdCl_3_ transforms into Gd_2_O_3_ above 1100 °C [53], while TbCl_3_ is almost completely converted to Tb_7_O_12_ above 1000 °C [86,87]. YCl_3_ begins to transition to Y_2_O_3_ at 800 °C, though a small amount of YOCl often persists. In summary, the minimum pyrolysis temperatures for the conversion of these seven rare-earth chlorides to their respective oxides increase in the following order: CeCl_3_ < YCl_3_ < TbCl_3_ < NdCl_3_ < GdCl_3_ < PrCl_3_ < LaCl_3_. The decomposition sequence of rare-earth chlorides with increasing temperature is illustrated in Figure 10.

2. O_2_ Partial Pressure

**Figure 11:**
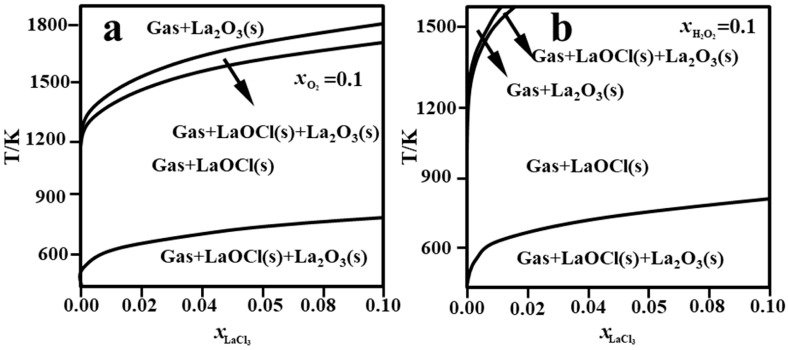
Phase diagram of lanthanum chloride pyrolysis: (**a**) without H_2_O_2_ addition; (**b**) with H_2_O_2_ addition [90].

The oxygen partial pressure in the system plays a significant role in determining the phase composition of products during the pyrolysis of rare-earth chlorides. At a fixed temperature, lower chloride concentrations and higher O_2_ partial pressures favor the formation of oxides [53]. For example, the phase diagram for the LaCl_3_-H_2_O_2_ system (Figure 11) shows that the addition of hydrogen peroxide markedly narrows the stability region of LaCl_3_. This effect is attributed to the strong oxidizing activity of H_2_O_2_ in aqueous solution, which promotes the pyrolysis of chloride precursors [52]. Under standard conditions, the presence of hydrogen peroxide substantially lowers the pyrolysis temperature of rare-earth chloride solutions. With the exception of Pr, all rare-earth chloride solutions can be converted to oxides at low temperatures ranging from 100 °C to 500 °C [59].

3. H_2_O and HCl Partial Pressure

**Figure 12:**
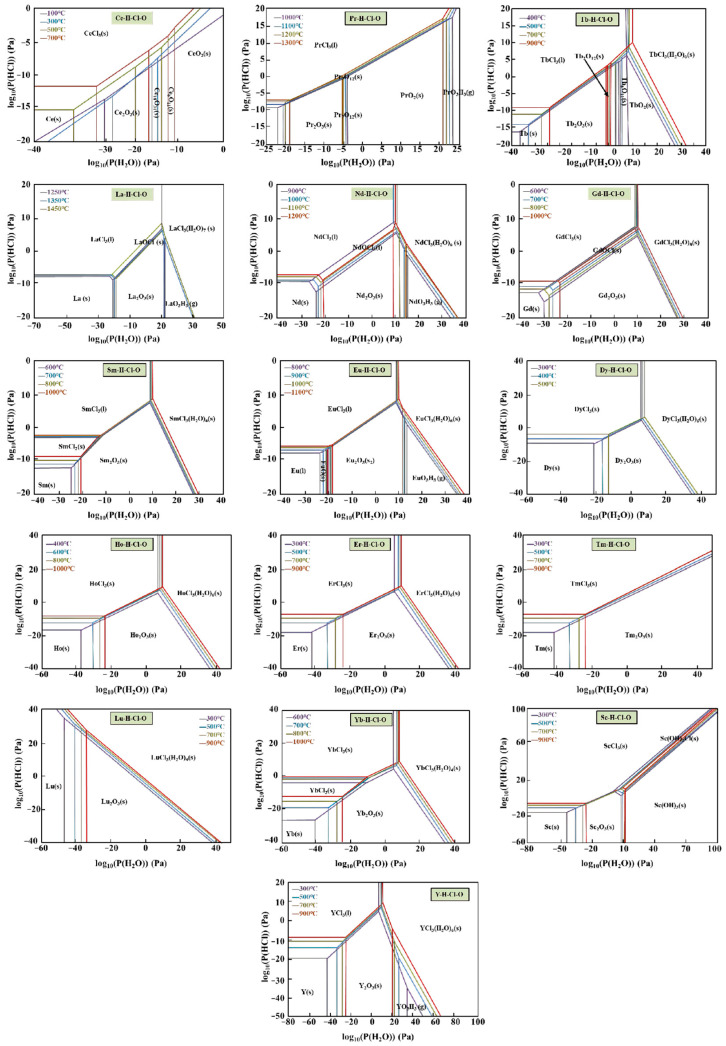
Predominance-area diagrams of the RE-H-Cl-O system as a function of temperature under various water vapor partial pressures (Generated using Factsage 8.2 software).

The thermal decomposition of metal chloride solutions into oxides proceeds via hydrolysis. Consequently, in addition to oxygen partial pressure, the partial pressures of H_2_O and HCl in the reaction system are also critical factors influencing the final oxide formation. Lowering the partial pressures of HCl and H_2_O can further reduce the initial reaction temperature [67]. Due to the varying temperatures required for intermediate and final product formation during the pyrolysis of different rare-earth chlorides, Figure 12 illustrates the favorable operating zones for pyrolysis of selected rare-earth chlorides. As revealed in Figure 12, when oxygen partial pressure is negligible and the partial pressures of both water vapor and HCl are close to 1 bar (i.e., $lgP_{H_{2}O}/P^{\theta }\approx lgP_{HCl}\approx 0$), REO products form across the entire temperature range. Taking SmCl_3_ as an example, under these conditions and within a temperature range of 900 °C to 1300 °C, Sm_2_O_3_ is produced.

### 3.2. Kinetic Mechanism of Pyrolysis

The pyrolysis kinetics of rare-earth chlorides are generally described using gas–solid reaction models. Among the light rare-earth chlorides, CeCl_3_, PrCl_3_, and NdCl_3_ have been the most extensively studied and are presented here as representative examples to illustrate general kinetic trends.

CeCl_3_ exhibits the lowest apparent activation energy (Ea), allowing decomposition at relatively low temperatures. In contrast, PrCl_3_ and NdCl_3_ possess higher Ea values, necessitating elevated temperatures for significant thermal decomposition [80]. The rate-limiting mechanisms also differ: CeCl_3_ decomposition is primarily governed by internal particle diffusion coupled with intrinsic chemical reaction, whereas PrCl_3_ and NdCl_3_ are controlled mainly by intrinsic chemical reaction kinetics [80]. Factors such as particle or droplet size significantly influence kinetic parameters, with larger sizes generally increasing Ea and decreasing reaction rates [78,79].

For clarity and conciseness, key kinetic parameters for these representative rare-earth chlorides under different experimental conditions are summarized in Table 2. Representative kinetic equations are also provided to illustrate typical reaction behaviors.

### 3.3. Phase-Transition Mechanism of Pyrolysis

**Figure 13:**
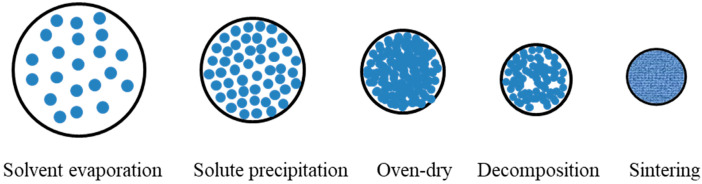
Schematic of the powder formation process in pyrolysis [21,44,45,46,47,48,49,50,81].

During the pyrolysis conversion of rare-earth chlorides into oxides, the transformation process of droplets inside the reactor is the most critical step, as it directly governs key performance indicators of the resultant powders [21]. Among these, particle morphology is largely determined by the evolution of metastable phases. Figure 13 illustrates the reaction pathway of a single droplet during its transit through the furnace.

**Figure 14:**
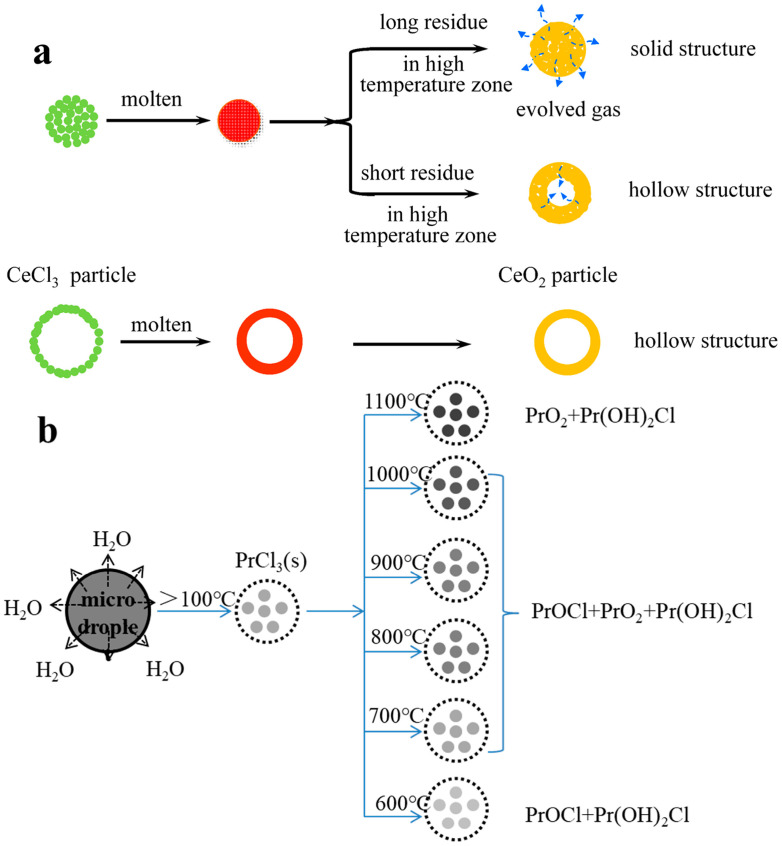
Schematic diagrams of the conversion mechanism from RECl_x_ to REO: (**a**) Ce [87]; (**b**) Pr [85].

The thermal decomposition of rare-earth chlorides proceeds through three consecutive stages: dehydration, hydrolysis, and oxide formation. Oxide formation occurs via two principal pathways: (i) direct oxidation of anhydrous chlorides to form REOs, and (ii) high-temperature oxidation of REOCl to yield the corresponding REO. Under pyrolytic conditions, variable-valence REEs do not form stable trivalent oxides; instead, they predominantly exist as mixed-valence oxides (Figure 14a,b). For example, CeCl_3_ pyrolysis primarily produces CeO_2_, whereas PrCl_3_ can form PrO_2_ under high-temperature oxidizing conditions. Depending on the reaction atmosphere, TbCl_3_ may yield Tb_7_O_12_ or TbO_2_. The phase transformation pathways for these rare-earth chlorides are summarized in Table 3.

## 4. Pyrolysis Reactors and Technology Applications

### 4.1. Pyrolysis Reactors

Building upon the preceding discussion of thermodynamic and kinetic principles, as well as the influences of process parameters on the pyrolysis of rare-earth chlorides, it is essential to translate these fundamental insights into practical reactor design and operation. The reactor configuration not only governs the efficiency and scalability of the pyrolysis process but also significantly influences product quality, including purity, particle size, and microstructure. Therefore, a clear understanding of the advantages and limitations of different reactor types is crucial for bridging theoretical knowledge and industrial implementation.

Currently, chloride pyrolysis reactors are primarily classified into static and dynamic types (including SP and JP) [93]. Early studies on chloride pyrolysis predominantly used static reactors such as sealed tube furnaces. Although structurally simple, these systems are limited by low productivity and high energy consumption due to their batch operation mode, making them poorly suited for industrial-scale application. In recent years, research has shifted toward continuous dynamic reactors. Among these, dynamic pyrolysis methods, characterized by continuous operation and one-step synthesis, offer broad prospects for industrial adoption [94].

**Figure 15:**
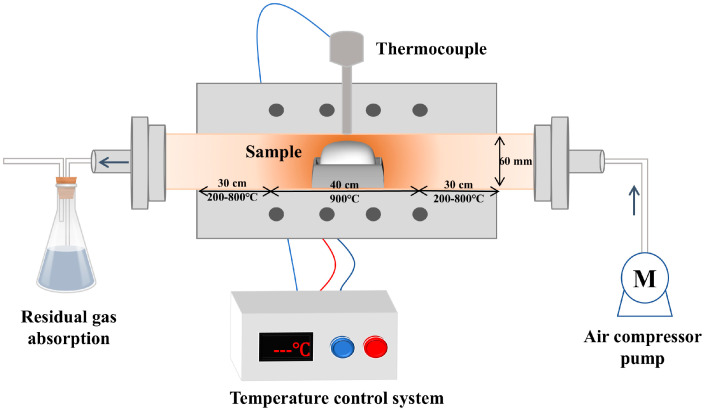
Experimental setup for static thermal decomposition.

Static pyrolysis typically employs sealed tube furnaces (Figure 15) to decompose crystalline rare-earth chlorides at specific temperatures. This approach is often used for initial exploration of reaction temperatures and decomposition mechanisms.

Dynamic pyrolysis uses rare-earth chloride solutions, either as bulk solutions or aerosols, as precursors to prepare nanoparticles via high-temperature decomposition. The properties of the resulting products are influenced by both the precursor composition and pyrolysis parameters. When required, complexing agents such as citric acid or hydrogen peroxide can be added to the precursor solution. By tailoring the precursor formulation and process conditions, functional nanomaterials with controlled size and morphology can be obtained. Based on the heating configuration, dynamic pyrolysis can be categorized into horizontal tube furnace SP, vertical tube furnace SP, and JP.

1. Horizontal Tubular Furnace Spray Pyrolysis (HTFSP)

Horizontal Tubular Furnace Spray Pyrolysis (HTFSP) is one of the most widely employed methods in dynamic chloride pyrolysis for synthesizing REOs. In this process, an ultrasonic nebulizer converts the chloride precursor solution into micron-sized droplets, which are transported by a carrier gas into a heated quartz or ceramic tubular furnace. Each droplet functions as an individual microreactor, undergoing sequential stages of solvent evaporation, solute precipitation, and solid-state decomposition within a residence time of only a few seconds.

**Figure 16:**
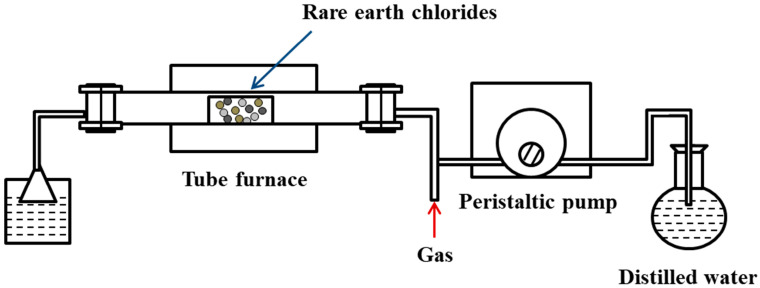
Schematic diagram of the HTFSP heating system [40].

The HTFSP system (Figure 16) typically comprises a carrier gas supply, a precursor solution reservoir, an atomizer, a tubular pyrolysis chamber, and a powder collector or substrate for thin-film deposition [10]. This configuration offers several advantages, such as continuous operation, high stability, efficiency, one-step synthesis, and low operational cost. However, it also presents certain drawbacks, including relatively low product crystallinity and low tap density of the resulting samples [10]. To improve crystallinity, additional post-treatment (such as calcination, oxidation, or reduction) is often applied to the collected solid powders or films.

2. Vertical Spray Pyrolysis (VSP)

**Figure 17:**
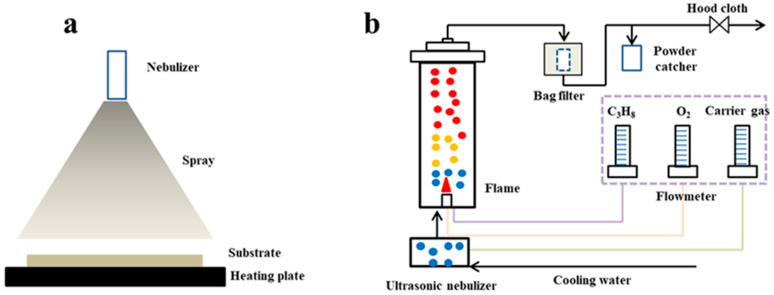
Schematic diagrams of vertical spray pyrolysis heating apparatus [99]: (**a**) SPD, (**b**) FSP.

Vertical Spray Pyrolysis (VSP) also referred to as “top-down” or “drop-tower” SP, positions the atomizer directly above a vertically oriented heated reaction tube. Compared with horizontal tubular furnaces, the gravity-assisted downward trajectory in VSP reduces droplet–wall interactions and narrows the residence time distribution, resulting in denser and more spherical particles [56]. VSP systems can be categorized into three main types:

Spray pyrolysis deposition (SPD, Figure 17a) is primarily employed for thin-film fabrication. In this method, the precursor solution is sprayed as an aerosol through a nozzle onto a heated substrate [95]. On the heated substrate, the droplets undergo evaporation, precipitation, drying, and decomposition, ultimately forming a thin film. By adjusting process parameters (e.g., nozzle-to-substrate distance, pyrolysis temperature, duration, and droplet rate) and the solution composition, the composition and morphology of the film can be precisely controlled [10].

Flame spray pyrolysis (FSP, Figure 17b) is predominantly used for synthesizing solid powders, including nano-metal oxides and doped metal oxides. This technique involves passing precursor-laden droplets through a flame, which triggers particle nucleation, growth, and coalescence into large solid particles that are subsequently collected by a bag filter [96,97,98]. However, FSP typically requires high temperatures, and the presence of oxygen makes it difficult to synthesize non-oxide phases. Moreover, the rapid reaction kinetics hinder control over nucleation and agglomeration, limiting the formation of metastable crystal phases [98].

**Figure 18:**
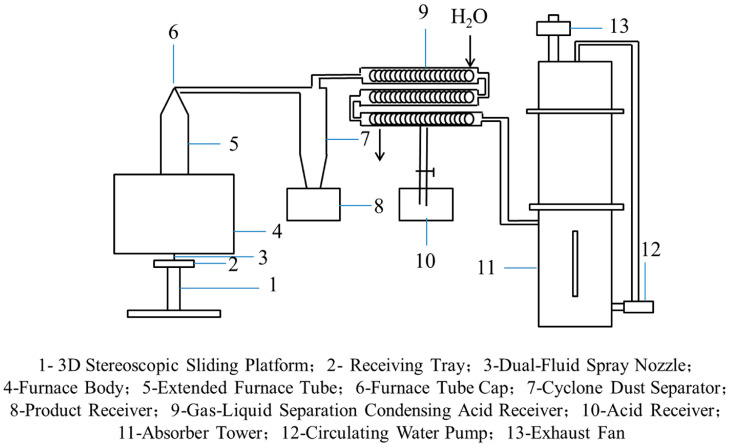
Schematic diagram of the vertical spray pyrolysis apparatus [52].

To extend the residence time of droplets in the high-temperature zone during VSP, a bottom-spray vertical reactor has been developed (Figure 18). In this configuration, the precursor solution is atomized into a mist or fine droplets via an atomizing nozzle or an ultra-fine high-pressure nozzle. The mist or droplets enter the vertical furnace from the bottom and are rapidly converted into oxide powders. When processing cerium chloride at a feed rate of 1.344 L/h, this reactor achieves a CeO_2_ yield of 88.7% and recovers HCl at a concentration of 7.08 mol/L. Simultaneous real-time monitoring of temperature and pressure is also achieved [100].

3. Jet Pyrolysis (JP)

**Figure 19:**
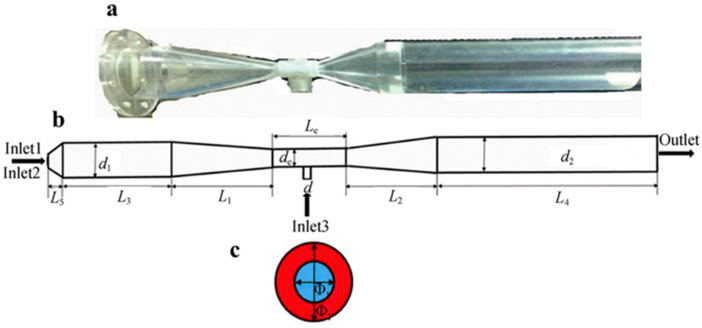
Jet-flow pyrolysis reactor: (**a**) real image; (**b**) dimension; (**c**) section of gas-phase inlet [104,105,106].

Jet Pyrolysis (JP) represents an advanced development of conventional SP. To further reduce the decomposition temperature required for rare-earth chlorides, improvements have been made to the SP apparatus [58,59]. Researchers have employed JP to investigate the decomposition behavior of CeCl_3_, PrCl_3_, and TbCl_3_. For example, Liu et al. [67] proposed a small-scale JP system with a production capacity of 300 g/h of lanthanum oxide. During pyrolysis, the by-product hydrochloric acid is recycled back into the rare-earth extraction process, enabling cleaner production from raw minerals to final REO products. Like SP, JP achieves rapid pyrolysis at lower temperatures, compared to traditional static pyrolysis in muffle furnaces [101]. In the case of CeCl_3_, JP produces pure CeO_2_ at 500 °C, with a residual chlorine content of 0.2 wt%, whereas static pyrolysis requires a temperature above 700 °C to initiate the reaction. JP also offers benefits such as lower energy consumption and reduced emissions of pollutants such as HCl, supporting the green and efficient preparation of high-purity REOs [58,59]. The JP reactor is based on a Venturi design (Figure 19), which provides a simple structure, a relatively uniform droplet size distribution, low production cost, and strong potential for industrial adoption [67]. Material and energy balance analyses can be applied to determine relevant process parameters [67]. In addition, detailed simulations have been conducted to study the JP device and the influence of various operating factors [102,103,104,105], facilitating its transition into an efficient, economical, and environmentally friendly technology with broad industrial applicability. Table 4 presents a comparison of the advantages and disadvantages of pyrolysis reactors.

**Figure 20:**
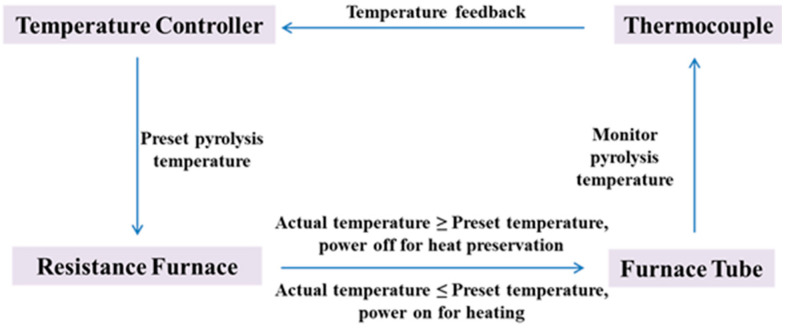
Logic diagram of the pyrolysis reactor temperature-control system [99].

The temperature-control strategy of the pyrolysis unit is illustrated in Figure 20. A set-point temperature is initially defined, after which the heating system activates the resistance furnace. A temperature sensor provides real-time feedback to the controller, enabling closed-loop control to maintain precise thermal conditions. This ensures that the reaction proceeds under optimal temperature conditions and supports systematic investigation of pyrolysis kinetics.

### 4.2. Technology Applications

The industrial application of direct pyrolysis is predominantly centered on spray pyrolysis technology. Initially, this technology was employed for waste acid recovery in the steel industry. During the 1980s, several major Chinese steel enterprises—including Anshan Iron and Steel Group Co., Ltd. (Anshan, China), Baoshan Iron and Steel Co., Ltd. (Shanghai, China), Pangang Group Co., Ltd. (Pangzhihua, China), and Hansteel Co., Ltd. (Shanghai, China).—adopted the Ruthner process technology from the Austrian company ANDRITZ (Graz, Austria) for the recovery and reuse of spent acid [24].

In recent years, researchers have utilized dynamic pyrolysis reactors, such as HTFSP and VSP, to achieve efficient treatment of acidic waste streams. In 2023, pilot-scale pyrolysis studies were conducted on magnesium chloride derived from salt lake brine, with a process model developed using Aspen Plus V11 software to support system design and optimization [107].

Currently, spray pyrolysis is primarily applied in key advanced material sectors, including the synthesis of semiconductor materials and energy storage materials. However, its widespread industrial adoption remains constrained by high initial capital investment. Although spray pyrolysis has demonstrated promising and consistent performance at the laboratory scale, scaling up to industrial production presents significant technical and economic challenges. Nonetheless, the global market for spray pyrolysis is expanding. According to market analysis, the global spray pyrolysis system market was valued at approximately 133 million USD in 2024 and is projected to reach 193 million USD by 2031, reflecting a compound annual growth rate of 4.7% [108].

Presently, spray pyrolysis is attracting increasing academic and industrial interest. Table 5 summarizes key companies involved in pyrolysis technologies, along with their respective methods and primary application domains. With growing recognition of its potential, investment in spray pyrolysis has significantly increased in recent years. Ongoing technological innovation holds promise for overcoming traditional limitations and enabling new applications. Furthermore, advancements in process monitoring and related technical methodologies [100] are expected to pave the way for further development and industrial integration of spray pyrolysis technology.

## 5. Conclusions

Spray pyrolysis-calcination is an advanced technique for synthesizing REOs. Compared to static pyrolysis, this method offers distinct advantages and has broad potential for industrial application. A comprehensive review and analysis of current research on rare-earth chloride pyrolysis led to the following conclusions:

(1) Mechanistic studies reveal that, in the absence of additives, the onset decomposition temperatures of rare-earth chlorides increase in the order CeCl_3_ < YCl_3_ < TbCl_3_ < NdCl_3_ < GdCl_3_ < PrCl_3_ < LaCl_3_. The introduction of additives such as citric acid or H_2_O_2_, coupled with elevated O_2_ partial pressure and reduced HCl partial pressure, significantly lowers the decomposition temperature. Compared with static pyrolysis, SP exhibits a lower apparent activation energy for rare-earth chloride decomposition, thereby promoting more efficient REO formation.

(2) The thermal decomposition of rare-earth chlorides proceeds through three consecutive stages: dehydration, hydrolysis, and oxide formation. Oxide formation occurs via two parallel pathways: (i) direct oxidation of anhydrous chlorides to REOs, and (ii) initial formation of REOCl followed by high-temperature oxidation to the corresponding oxides. Under pyrolytic conditions, variable-valence REEs do not form stable trivalent oxides but instead stabilize as mixed-valence phases.

(3) Pyrolysis reactors for rare-earth chlorides can be classified into horizontal tube furnaces, vertical tube furnaces, and JP reactors. Among these, vertical tube configurations have the highest industrial relevance. Reactor design must be optimized according to specific decomposition temperatures and chloride concentrations, while also considering energy efficiency and acid recovery.

(4) Although SP has demonstrated excellent performance in producing both high-purity single and composite REOs, its application remains largely confined to the laboratory scale, with limited industrial implementation reported to date. Moreover, systematic studies on the pyrolysis mechanisms and process conditions of medium- and heavy-rare-earth chlorides are still lacking. The scale-up of SP technology faces considerable challenges, including stringent equipment requirements, operational complexity, demands for high-purity feedstocks, and issues related to residual chlorine content. Addressing these challenges is essential for the successful industrial adoption of SP-based REO production.

## Figures and Tables

**Table 1:** Overview of pyrolysis processes and product performance.

Rare-Earth Elements		Material Compositions	Preparation Process	Product Features	References
Constant—Valence Rare-earths	La	AlCl_3_·6H_2_O, >99.0%; LaCl_3_·7H_2_O, >99.0%; C_2_H_5_OH, >99.0%	Temperature: 800 °C; Time: 2 h; Concentration: 20 wt%	LaAlO_3_ purity > 99.9%; LaOCl and Al_2_O_3_ particle size: 0.5–15.0 μm; hollow spheres and plate-like particles.	[68,69,70,71]
		LaCl_3_ crystals, >99%; LaCl_3_, 400 g/L; Additives: H_2_O_2_(AR)	Temperature: 600 °C; Carrier gas pressure: 0.4 MPa; H_2_O_2_(AR): 5%	Primary product is lanthanum hydroxide; conversion rate 99.96%; fragmented particle morphology.	[52]
		LaCl_3_(Industrial grade), 0.4 mol/L; H(OCH_2_CH_2_)_n_OH (molecular weight 20,000, AR); NaOH(AR)	608–750 °C→La(OH)_3_; 750 °C (2 h)→La_2_O_3_	Specific surface area: 36.54 m^2^/g; equivalent diameter: 25.22 nm.	[64]
	Gd	GdCl_3_, 347.41 g/L; Additives: C_6_H_8_O_7_·H_2_O, ≥99.5%	Temperature: 750 °C; Time: 2 h	Approximately spherical or fragmented morphology; chlorine content < 500 ppm.	[53]
	Nd	NdCl_3_, 207.75 g/L; C_6_H_8_O_7_·H_2_O, ≥99.5%	Temperature: 1050 °C; Time: 4 h	Particle size: 1.929–3.830 μm; loose, porous spherical particles with flaky surface; Cl^−^ content well below 500 ppm.	[53]
Variable—Valence Rare-earth	Ce	CeCl_3_·7H_2_O, 100 g/L	Temperature: 600 °C	Hollow spherical particles with regular morphology, clear boundaries, good dispersibility; particle size: 0.06–3.28 μm; polycrystalline nanocrystals with small grain sizes.	[76]
		CeCl_3_, 20 wt%	Temperature: 650 °C; Jet velocity: 10 m/s; Ambient pressure: 1 atm	Smooth, solid spheres; narrow size distribution: 0.5–1 μm; high purity.	[77]
		CeCl_3_·7H_2_O, 30 wt%; C_6_H_8_O_7_·H_2_O, 99.5%	Pressure 0.3 MPa; Temperature: 650 °C; Time: 2 h; AR:CeCl_3_(molar ratio): 1.5	Minimum particle size approximately 15 nm; agglomerated nanoparticles; maximum specific surface area 59.72 m^2^/g; well-crystallized; uniformly distributed spherical polycrystals; high purity.	[78]
	Pr	PrCl_3_, 65.73 g/L; C_6_H_8_O_7_·H_2_O, ≥99.5%	Temperature: 1050 °C; Time: 4 h	Particle size: 1.929–3.830 µm; loosely porous spherical particles with lamellar surface; Cl^−^ content well below 500 ppm.	[53]
	Tb	TbCl_3_·6H_2_O, 0.05 mol/L	Carrier gas flow rate; 10 L/min; Temperature: 600 °C	Uniform spherical particles with regular morphology; particle size: 0.1–1.3 μm; d_50_ approximately 0.5 μm; high specific surface area.	[86,87]

**Table 2:** Comparative kinetic parameters of CeCl_3_, PrCl_3_, and NdCl_3_ under different experimental conditions.

Compounds	Conditions	Ea (kJ/mol)	Rate-Limiting Mechanisms	Kinetic Equations	References
CeCl_3_	Static	25.836	Internal diffusion control	1 − (2/3)a − (1 − a) = 0.706exp(25.836/RT)t	[80,91]
	Dynamic	22.431	Mixed-control (diffusion + reaction)	1 − (2/3)α − (1 − α)^(2/3) = 0.646exp(−22.431/RT) t.	[92]
	Droplet size effect (V = 2–10 μL)	36–62.5	Mixed-control (diffusion + reaction)	(0.627–0.012)exp(Ea/RT) t	[78,79]
	DSC-TG	33.914	Chemical reaction	-	[80]
PrCl_3_	Static	132.479	Chemical reaction	1 − (1 − α)^(1/3) = 664.390exp(−132.479/RT) t	[80]
	DSC-TG	107.04	Chemical reaction	-	[80]
NdCl_3_	Static	181.544	Chemical reaction	1 − (1 − α)^(1/3) = 21451exp(−181.544/RT) t	[80]
	DSC-TG	107.22	Chemical reaction	-	[80]

Note: DSC-TG in Table 2 refers to Differential Scanning Calorimetry-Thermogravimetric Analysis.

**Table 3:** Phase transformation pathways during pyrolysis of rare-earth chlorides.

REEs	Phase Transformation Pathway
Pr	PrCl_3_ → Pr(OH)_2_Cl → PrOCl → Pr_6_O_11_ → Pr_2_O_3_
Ce	CeCl_3_ → CeO_2_
Tb	TbCl_3_ → Tb(OH)_2_Cl → TbOCl → TbO_2_ → 2Tb_7_O_12_
	TbCl_3_ → Tb(OH)_2_Cl → TbOCl → 2Tb_7_O_12_
Sm, Eu	RECl_3_ → RE_2_O_3_
Gd, Nd	RECl_3_ → RE (OH)_2_Cl → REOCl → RE_2_O_3_

**Table 4:** Reactor Types in Rare-Earth Chloride Pyrolysis.

Reactor Types	Advantages	Disadvantages	References
Static Pyrolysis (Sealed Tube Furnace)	Simple structure; easy operation; suitable for small-scale research.	Low productivity; high energy consumption; batch operation limits industrial scalability.	[93]
HTFSP	Continuous operation; high efficiency; one-step synthesis; low cost; stable.	Low crystallinity and tap density; requires post-treatment (calcination, oxidation, reduction).	[10]
VSP	Gravity-assisted trajectory reduces wall interactions; denser particles.	Limited control over nucleation/agglomeration; high temperatures required in some configurations.	[56,95]
FSP	Suitable for nano-metal oxides and doped oxides; rapid synthesis.	High temperature required; difficult to synthesize non-oxides; poor control over agglomeration.	[96,97,98]
SPD	Enables thin-film fabrication; high control over composition and morphology.	Limited to film deposition; potential non-uniformity issues.	[95]
JP	Low decomposition temperature; clean production; low energy consumption; low emissions.	Scalability requires optimization; parameter sensitivity in large-scale operation.	[58,67,101]

**Table 5:** Major Companies in Spray Pyrolysis at Present [108].

Companies	Spray Methods	Main Application Areas
Holmarc Opto-Mechatronics Pvt. Ltd. (Kerala, India)	Ultrasonic spray	Research in thin films; Coating
Sono-Tek Corporation (New York, USA)	Ultrasonic atomization	Semiconductor coating
Siansonic Technology Co., Ltd. (Beijing, China)	Ultrasonic spray	Precision spraying; spray pyrolysis; spray drying
Sonaer Inc. (New York, USA.)	High-throughput spray	Semiconductor
Zhengzhou CY Scientific Instrument Co., Ltd. (Zhengzhou, China)	Ultrasonic spray	Spray Pyrolysis Coating System

## Data Availability

No new data were created or analyzed in this study. Data sharing is not applicable to this article.
